# Gelation Kinetics of Hydrogels Based on Acrylamide–AMPS–NVP Terpolymer, Bentonite, and Polyethylenimine for Conformance Control of Oil Reservoirs

**DOI:** 10.3390/gels5010007

**Published:** 2019-02-14

**Authors:** Fernanda G.C. Tessarolli, Sara T.S. Souza, Ailton S. Gomes, Claudia R.E. Mansur

**Affiliations:** Institute of Macromolecules (IMA), Federal University of Rio de Janeiro (UFRJ), Av. Horácio Macedo, 2030, Ilha do Fundão, Rio de Janeiro CEP 21941-598, RJ, Brazil; sara.talita1207@gmail.com (S.T.S.S.); asgomes@ima.ufrj.br (A.S.G.); celias@ima.ufrj.br (C.R.E.M.)

**Keywords:** composite hydrogel, AMPS–NVP–acrylamide copolymer, conformance control, oil reservoir

## Abstract

Relatively smaller volumes of gelling systems had been used to address conformance problems located near the wellbore in oil reservoirs with harsh temperature and salinity conditions. These gelling systems were formulated with high concentrations of low-molecular-weight acrylamide-based polymers crosslinked with polyethylenimine (PEI). However, for in-depth conformance control, in which large gelant volumes and long gelation times were required, lower-base polymer loadings were necessary to ensure the economic feasibility of the treatment. In this study, a gelling system with high-molecular weight 2-acrylamido-2-methylpropane sulfonic acid (AMPS), N-vinyl-2-pyrrolidone (NVP), acrylamide terpolymer, and PEI, with the addition of bentonite as a filler, was formulated. The influence of the gelant formulation and reservoir conditions on the gelation kinetics and final gel strength of the system was investigated through bottle tests and rheological tests. The addition of clay in the formulation increased the gelation time, thermal stability, and syneresis resistance, and slightly improved the final gel strength. Furthermore, samples prepared with polymer and PEI concentrations below 1 wt %, natural bentonite, and PEI with molecular weight of 70,000 kg/kmol and pH of 11: (i) presented good injectivity and propagation parameters (pseudoplastic behavior and viscosity ~25 mPa·s); (ii) showed suitable gelation times for near wellbore (~5 h) or far wellbore (~21 h) treatments; and (iii) formed strong composite hydrogels (equilibrium complex modulus ~10–20 Pa and Sydansk code G to H) with low syneresis and good long-term stability (~3 to 6 months) under harsh conditions. Therefore, the use of high-molecular-weight base polymer and low-cost clay as active filler seems promising to improve the cost-effectiveness of gelling systems for in-depth conformance treatments under harsh conditions of temperature and salinity/hardness.

## 1. Introduction

Conformance control is a permeability-reducing treatment that can be applied to decrease or eliminate an oil recovery conformance problem in the wellbore or within the reservoir located near to or far from the wellbore (distances > 15 m). The problems include: (i) excessive and competing water or gas production emanating from a casing leak or flow behind the pipe; (ii) poor sweep efficiency and/or excessive co-production of the oil recovery drive fluid in a matrix-rock reservoir resulting from substantial permeability variation or anomalies (e.g., fractures); and (iii) water or gas from an aquifer or gas cap coning up/down the producing interval of a vertical well through the matrix rock or anomaly [1].

Several polymer gelling systems have been developed as diverting and blocking agents to remedy well and reservoir conformance problems. The screening and selection of the most appropriate system for a given conformance treatment should take into consideration the reservoir conditions (temperature, salinity, hardness, and pH value); technical aspects (injectivity, propagation, penetration, strength, and stability requirements); economic aspects (volumes, concentrations, and shut-in times); and environmental legislation requirements. Other parameters to be considered include compatibility with other chemicals; the salinity and the pH value of water used to prepare the systems and the formation water; the permeability of the target zone; the lithology of the formation; and the presence of either carbon dioxide (CO_2_) or hydrogen sulfide (H_2_S) [2,3].

Organically crosslinked polymer (OCP) systems based on polyacrylamide homopolymer (PAM) and acrylamide copolymers, such as partially hydrolyzed polyacrylamide (PHPA), poly(acrylamide-*co*-*t*-butyl acrylate) (PAtBA), poly(acrylamide-*co*-2-acrylamido-2-methyl-propanesulfonic acid) (PAM-AMPS), and poly(acrylamide-*co*-*N*-vinyl-2-pyrrolidone) (PAM-NVP), crosslinked with polyethylenimine (PEI), had been applied for the conformance treatment of wells and reservoirs with unfractured matrix rock or anomalies (e.g., single fractures or network fractures) with harsh conditions of temperature (>80 °C) and salinity/hardness (>30,000 mg/L total dissolved solids (TDS)) [4,5,6,7].

The OCP systems were prepared at the surface facilities and pumped as low-viscosity gelants (polymer + crosslinking agent solutions) into the well or reservoir around the wellbore, where they selectively flow through the high-permeability zones or anomalies. After sufficient time, a stable three-dimensional structure (chemical hydrogel) was formed in situ by means of the nucleophilic attack of the amine nitrogens of the PEI on the carbonyl carbons of the amide groups of the polyacrylamide (transamidation reactions). Thus formed hydrogel behaved as a flow diverting or blocking agent within the preferential paths existing in the well or reservoir [8,9].

For conformance control of oil reservoirs, these gelling systems must have good injectivity and propagation within the reservoir rock (viscosities below 30 mPa·s); predictable and controllable gelation time (>2 h to ensure safety and operational continuity); and long-term biological, chemical, and thermal stability [6,10]. Furthermore, the gelant must form a strong hydrogel under reservoir conditions—with Sydansk gel-strength code > G, and storage modulus (G’) and complex modulus (G*) preferably >10 (blocking-ability parameters)—in order to withstand the pressure gradients and water flow expected in the formation [11,12,13].

The initial viscosity, gelation time, and final gel strength of these systems depended on the components of the gelant (polymer, crosslinker, and solvent); the concentration of the reagents; the presence of additives; and the reaction conditions (pH value, salinity/hardness, and temperature) [14,15].

Typically, acrylamide-based OCP systems were formulated with high polymer (5 to 7 wt %) and crosslinker (1 to 2 wt %) concentrations to form stiffer hydrogels, and low-molecular-weight base polymers to assure low-surface fluid viscosities (<30 mPa·s) [1,4,16].

However, for in-depth conformance treatment of reservoirs with harsh conditions (high temperature and high salinity/hardness), long gelation times (>8 h) were required. Therefore, any effort to improve the cost-effectiveness of OCP systems required the reduction of the base polymer loading in the treatment fluid, which had the associated positive effect of lengthening the gelation time of the formulation, but the negative impact of reducing the strength of the hydrogels formed [17,18].

Formulating acrylamide-based OCP systems with high-molecular-weight polymer (>1,000,000 kg/kmol) allowed significant reduction in the base polymer concentration (≤1 wt %) [4]. Moreover, the presence of 2-acrylamido-2-methylpropane sulfonic acid (AMPS) and *N*-vinyl-2-pyrrolidone (NVP) groups in acrylamide-copolymer chains improved the shear resistance, salt-tolerance, and/or prevented the acrylamide groups from autohydrolyzing at higher temperatures (≥70 °C), reducing the polymer’s tendency to precipitate out of the solution in the presence of divalent ions (e.g., Ca^2+^ or Mg^2+^), making them more favorable for high salinity/hardness applications than acrylate–acrylamide copolymers [19,20,21,22]. Also, the addition of lamellar clays to gelling formulations had been used to reduce the final cost of systems (filler), to increase the thermal resistance and mechanical strength (elastic modulus), as well as to reduce the syneresis and sensitivity to salinity of the (nano)composite hydrogels formed [23,24,25,26,27].

Therefore, the objective of this study was to investigate the effect of different parameters on the viscosity, gelation kinetics, and final gel strength of acrylamide–AMPS–NVP terpolymer, bentonite, and PEI systems through visual consistency testing (bottle tests) and dynamic shear tests. These parameters included (i) the gelant components, such as polymer concentration, crosslinker concentration, crosslinker properties (molecular weight and initial pH value), clay type (polycationic, sodium or organically modified), and clay concentration; as well as (ii) the reservoir conditions, such as temperature and salinity (total dissolved salt and salt type).

## 2. Results and Discussion

### 2.1. Characterization of the Polymer Samples

**Figure 1:**
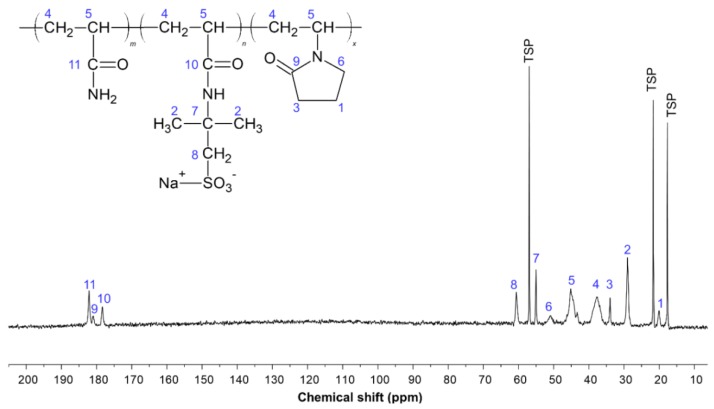
^13^C-NMR spectrum of the 2-acrylamido-2-methylpropane sulfonic acid (AMPS)–*N*-vinyl-2-pyrrolidone (NVP)–acrylamide terpolymer.

Figure 1 shows the ^13^C-NMR spectrum of the AMPS–NVP–AM terpolymer. The assignment of the peaks of the sample (Table 1) is in agreement with chemical shifts reported in the literature for acrylamide-based copolymers [15,28,29,30,31,32,33,34].

The chemical shifts and peak areas (A) of the carbonyl carbons were used to determine the acrylamide (AM), AMPS, and NVP moieties of the polymer samples (Equations (1)–(3), respectively).

(1) $$\%AM=\frac{A_{CONH_{2}}}{A_{CONH_{2}}+A_{AMPS}+A_{NVP}}\times 100$$

(2) $$\%AMPS=\frac{A_{AMPS}}{A_{CONH_{2}}+A_{AMPS}+A_{NVP}}\times 100$$

(3) $$\%NVP=\frac{A_{NVP}}{A_{CONH_{2}}+A_{AMPS}+A_{NVP}}\times 100$$

Table 2 shows the AM, AMPS, and NVP moieties determined by ^13^C-NMR, and the weight average molecular weight ($\bar{M_{w}}$) and polydispersion (PD) assessed by gel permeation chromatography (GPC) of the polymer.

### 2.2. Evaluation of the Injectivity and Propagation of the Gelling Systems

The initial consistency and the viscosity profiles of the gelants prepared in injection water were used to evaluate the injectivity and propagation capacity of the systems within reservoir rocks. According to the Sydansk visual code [11,35], all gelants prepared in oilfield brines presented liquid-like behavior at 25 °C and were classified with code ≤ B. Even though important, these preliminary static visual measurements (bottle tests) did not appropriately reflect how the gelling systems behaved in various shear regimes during conformance treatment, such as (i) during preparation in the surface facilities and injection through the tubing from the surface to the formation (usually coil tubing with reduced cross section), where shear rates above 1,000 s^−1^ could be found (i.e., mixing devices and pumps); and (ii) during propagation in the perforations and formation, where shear rates lower than 20 s^−1^ were generally found [36]. Therefore, it is important to understand how the viscosity of the gelling systems is affected by different shear rates, and how the viscosity of the gelant varies at a constant shear rate typically found in oil reservoirs, in order to evaluate, respectively, the injectivity and the propagation capacity of the formulations.

**Figure 2:**
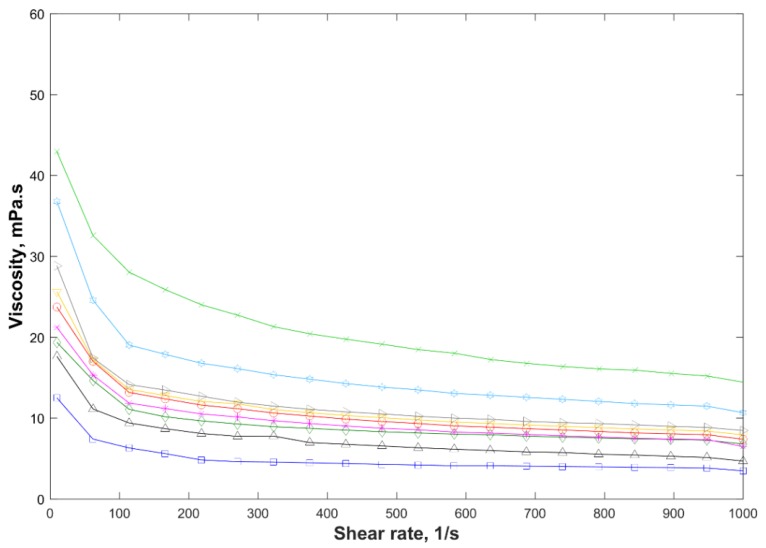
Return curves of the apparent viscosity versus shear rates for gelling systems prepared in desulfated seawater (injection water) at 25 °C. 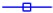 F1, 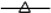 F2, 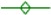 F3, and 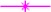 F4 with polyethylenimine (PEI) pH 7; 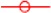 F4, 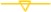 F5, 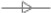 F6, and 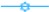 F4 with PEI 750,000 kg/kmol and 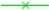 F7.

According to the experimental results, all gelants behaved as pseudoplastic non-Newtonian fluids under the conditions analyzed (Figure 2).

At rest, the gelling systems offered resistance to flow, mainly due to hydrogen bonding, entanglement of the polymer and crosslinker chains, and intra and intermolecular electrostatic repulsions promoted by the ionized sulfonate groups (–SO_3_^−^), which led to the unfolding of the base polymer chains and improved the dispersion of the negatively charged clay particles. As the shear rate increased, there was a reduction of the apparent viscosity of the systems due to the disentanglement and alignment of the polymer and crosslinker chains and clay tactoids in the flow direction. Such behavior was reversible and the gelling systems recovered their original viscosity when the applied shear was reduced or ceased. At higher shear rates, when the disentanglement and alignment of the polymer and PEI chains and clay tactoids in the flow direction were maximum, the gelling systems tended asymptotically to a constant and defined apparent viscosity value.

This moderate pseudoplastic behavior is extremely desirable to ensure good injectivity of gelants in conformance-improvement treatments. Near the wellbore, where high shear rates were found, the lower viscosity of pseudoplastic fluids reduced the fluid flow resistance, and thus decreased the power required for their injection into the reservoir. Away from the wellbore, where small average shear rates were found, a moderate increase in the fluid viscosity assisted the oil displacement in the reservoir without creating an additional flow resistance.

As expected, the shear viscosity of the gelants decreased at neutral pH values, and increased with rising polymer concentration, crosslinker concentration, molecular weight, and/or clay concentration due to (i) the increase in the polymer and PEI entanglements; (ii) the inter- and intramolecular electrostatic repulsion between the anionic sulfonate charged groups (–SO_3_^−^) that uncoiled the polymer chains, and the negative surface of the clay particles, which improved the particle dispersion; (iii) the adsorption of the polymer and the crosslinker on the clay particles by means of hydrogen bonds between the protons of the amide groups (–CONH_2_) of PEI and acrylamide, as well as the carbonyl oxygen of the NVP, and the silanol (–Si–OH) and aluminol (–Al–OH) groups of the clay platelets; and (iv) the clay/clay tactoid interactions [37,38,39].

In addition, all formulations except F4, prepared with PEI 750,000 kg/kmol, and F7, presented apparent viscosity between 10 and 30 mPa·s at a constant shear rate of 7.3 s^−1^ (Figure 2); these values recommended to ensure good propagation of the gelling systems in porous media [4,6,40,41]. Therefore, the polymer concentration and molecular weight of the crosslinker were the parameters that most strongly affected the viscosity of the gelants.

### 2.3. Influence of the Gelant Components and Reservoir Conditions on the Gelation Time and Gel Strength

The influence of the gelant components and reservoir conditions on the gelation kinetics and final gel strength of acrylamide–AMPS–NVP terpolymer, bentonite, and PEI systems was assessed by varying individually each parameter, while keeping the others constant.

#### 2.3.1. Polymer Concentration

**Figure 3:**
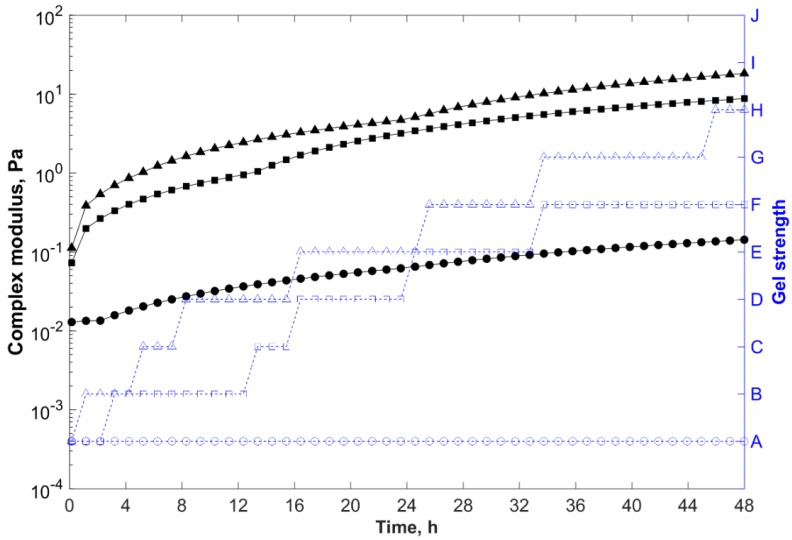
Complex modulus (─) and gel strength (- -) versus time for samples prepared in field water at 85 °C, with 0.8 wt % of clay, 0.5 wt % of PEI, and different polymer concentrations. 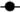

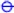
, 0.2 wt % (F1); 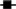

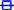
, 0.6 wt % (F4); and 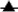

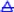
, 1.0 wt % (F7).

Gelants with higher polymer concentration presented shorter gelation times and greater storage modulus, and thus complex modulus (final gel strength) (Figure 3). As the polymer concentration increased in the gelling system, so did the intermolecular entanglements, hydrogen bonds between the polymer chains and clay particles, and intra and intermolecular electrostatic repulsions between sulfonate groups of AMPS (–SO_3_^−^) and newly charged carboxylate groups (–COO^−^) formed by the thermal hydrolysis of the acrylamide at temperatures above 80 °C, which uncoiled the polymer chains and improved the dispersion of the negatively charged particles within the gelant. As a result, more crosslinking sites of the polymer chains (acrylamide groups) became accessible to react with PEI, reducing the gelation time and increasing the final gel strength.

As previously discussed, even though the system prepared with a polymer concentration of 1.0 wt % (F7) showed gelation time around 5 h and formed a strong gel after 48 h (with Sydansk code = H and G*_e_ ~ 18 Pa), this formulation was considered unsuitable for in-depth conformance treatments due to the high viscosity at low shear rates (~50 mPa·s). Nevertheless, the reduction of the base polymer concentration from 1.0 wt % (F7) to 0.6 wt % (F4) decreased the viscosity of the gelant to 24 mPa·s, increased the gelation time to 13 h, and changed the quality of the gel from a stiff to a highly deformable type (with Sydansk code = F and G*_e_ ~ 9 Pa), decreasing the blocking ability of the gelling system.

Therefore, greater polymer loadings were required to formulate stronger composite hydrogels. However, higher polymer concentrations resulted in higher viscosities (limiting the injectivity and propagation of the systems) and shorter gelation times. Thus, for in-depth conformance-improvement treatments, in which low viscosities and longer gelation times were required, these opposing effects must be properly balanced [42,43].

#### 2.3.2. Crosslinker Concentration

**Figure 4:**
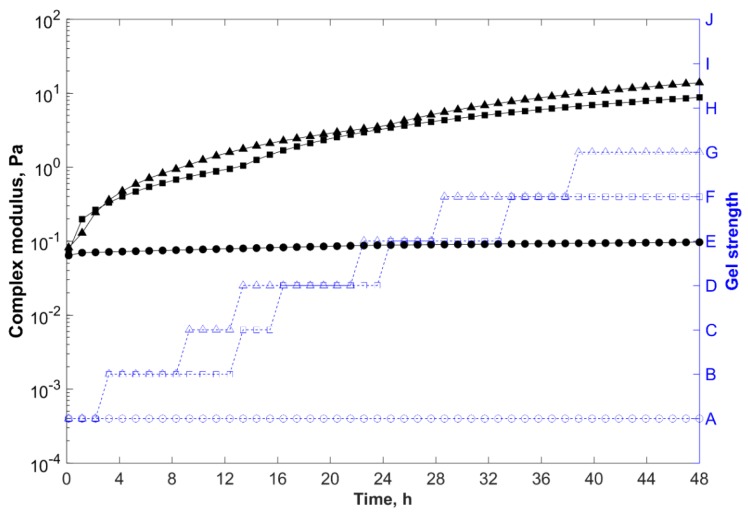
Complex modulus (─) and gel strength (- -) versus time for samples prepared in field water at 85 °C, with 0.6 wt % polymer, 0.8 wt % clay, and different PEI concentrations. 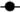

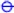
, 0.0 wt % (F2); 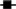

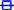
, 0.5 wt % (F4); and 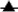

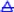
, 1.0 wt % (F6).

Gelants with higher PEI concentration presented shorter gelation times and greater storage modulus, and thus complex modulus (final gel strength) (Figure 4). As the crosslinker concentration increased in the gelling system, a greater number of crosslinking sites became available to react with the acrylamide groups of the polymer chains, reducing the gelation time and increasing the final gel strength.

With formulation F4 as reference, when the crosslinker concentration increased twofold to 1.0 wt % (F6), the viscosity of the gelant increased to 29 mPa·s and the gelation time decreased to 9 h. However, the system formed a deformable non-flowing gel after 48 h (with Sydansk code = G and G*_e_ ~ 14 Pa).

Nevertheless, the growth rate of the equilibrium complex modulus (G*_e_) promoted by the increase of the crosslinker concentration was less pronounced than the rate provided by the increase in the polymer concentration, and the final strength of the composite hydrogels was mainly determined by the concentration of polymer in the formulation rather than the concentration of PEI [16,44].

Furthermore, when PEI was not added, as in formulation F2, or the concentration of the crosslinker was too low (<0.1 wt %), the gelation did not take place and the system presented liquid-like behavior (Sydansk code = A and G*_e_ ~ 0.1 Pa). However, this does not mean that the higher the crosslinker concentration, the better the gel performance will be.

Adding crosslinker in excess may lead to over-crosslinking of the polymer chains, and consequently can cause syneresis of the hydrogels, reducing the blocking effectiveness of the gel treatment [42,45,46].

In this study, gelling systems with crosslinker concentration above 1.0 wt % presented syneresis after 30 days. Therefore, to achieve long-term stability, it is very important to use suitable crosslinker concentrations in the formulation of gelling systems [43,47].

#### 2.3.3. Crosslinker Molecular Weight

Gelants prepared with PEI with higher molecular weight presented shorter gelation times and greater storage modulus, and thus complex modulus (final gel strength). As the molecular weight of the crosslinker increased, more crosslinking sites became available to react with the acrylamide groups, reducing the gelation time and increasing the final gel strength.

With formulation F4 prepared with PEI with 70,000 kg/kmol as reference, when the molecular weight of the crosslinker increased tenfold to 750,000 kg/kmol, the viscosity increased to 37 mPa·s and the gelation time decreased to 9 h. However, the system formed a strong gel after 48 h (with Sydansk code = G and G*_e_ ~11 Pa). On the other hand, when the molecular weight of the crosslinker was 10,000 kg/kmol, the viscosity decreased to 21 mPa·s, the gelation time increased to 16 h, and formed a highly deformable gel after 48 h (with Sydansk code = F and G*_e_ ~ 5 Pa).

The growth rate of the equilibrium complex modulus (G*_e_) promoted by the increase in the molecular weight of the crosslinker was less pronounced than the rate caused by the increase in the crosslinker concentration. Furthermore, the addition of the crosslinker with a higher molecular weight caused syneresis of the hydrogels after 30 days at lower concentrations (above 0.5 wt %). Therefore, the crosslinker molecular weight can be used to fine tune the gelation kinetics and final gel strength of gelant formulations.

#### 2.3.4. Crosslinker Initial pH Value (Gelant pH Value)

During the preparation of the systems, we observed that the initial pH of the crosslinker controlled the pH value of the formulation. For instance, samples prepared with PEI having a pH value of 11 presented pH values around 11. Therefore, the discussion that follows applies to both effects—the initial pH value of PEI and the initial pH value of the gelling system.

The initial pH value of PEI or of the gelant changed the gelation kinetics and the final gel strength significantly. Gelants prepared with PEI with initial acid or alkaline pH values presented shorter gelation times and greater storage modulus, and thus complex modulus (final gel strength) than formulations prepared with PEI having the initial pH of 7.

With formulation F4 prepared with PEI with the initial pH value of 11 as reference, when the pH value was around 7, the viscosity was slightly reduced to 21 mPa·s, the gelation time increased to ~ 21 h, and the final gel strength was substantially reduced to G*_e_ ~ 1 Pa (with Sydansk code = C). In contrast, when the initial pH value of PEI was around 2, the viscosity increased to 23.5 mPa·s, the gelation time decreased to ~12 h, and the final gel strength declined to G*_e_ ~ 5 Pa (with Sydansk code = E). In contrast, when the initial pH value of PEI was around 2, the viscosity increased to 23.5 mPa·s, the gelation time decreased to ~12 h, and the final gel strength declined to G*_e_ ~ 5 Pa (with Sydansk code = E). When the initial pH value of PEI was around 9, the viscosity increased to 22 mPa·s, the gelation time decreased to ~15 h, and the final gel strength declined to G*_e_ ~ 6 Pa (with Sydansk code = F).

In acid or alkaline conditions, the hydrolysis rate of the amide groups of the terpolymer was greater than that at the pH value of 7 [42]. Hence, the intra and intermolecular electrostatic repulsion between the newly charged carboxylate groups (–COO^−^) and the existing sulfonate groups (–SO_3_^−^) uncoiled the polymer chains. As a result, the acrylamide groups became more accessible to the crosslinker molecules, reducing the gelation time and increasing the final gel strength.

Furthermore, the formation of stable three-dimensional structure (chemical hydrogel) by means of transamidation reactions (covalent bonding between the crosslinker and acrylamide-based polymer chains) depended on the initial nucleophilic attack of the amine nitrogen of the PEI on the carbonyl carbon of the amide groups of the polyacrylamide [8,9].

Nevertheless, according to Man [48], around 73%, 50%, 33%, 25%, and 4% of the unshared electron pairs of the nitrogen of the primary, secondary, and tertiary amino groups of the PEI were protonated at pH values of 2, 4, 5, 8, and 10, respectively. Thus, the nucleophilicity of PEI with acid-neutral pH values significantly decreased, making it less effective in crosslinking the acrylamide-based terpolymer, which led to embrittlement of the hydrogels (gel breakage). These results were in accordance with other findings reported in the literature [8,49].

Moreover, the protonation of PEI under acid or neutral pH values created positive charges along the polymer backbones. The intra- and intermolecular electrostatic repulsions among the charged groups promoted the extension of the crosslinker chains, favoring the adsorption of the PEI chains onto the negatively charged clay surfaces (even though the clay edges were positively charged), and the agglomeration of clay particles through the neutralization of the charged sites.

On the other hand, under alkaline conditions there were insufficient positive charges on the polymer backbone of PEI to keep the molecules extended through inter- and intrachain electrostatic repulsions, and both the edges and faces of the clay platelets carried negative charges. The lack of positive charges and the more compact conformation of the polymer chains reduced the adsorption of PEI onto the negatively charged clay particles, disfavoring the flocculation of the clay. Thus, more amino groups of the crosslinker were effectively available to react with the acrylamide-based copolymer chains, increasing the final gel strength [50].

In this study, we observed that PEI with a pH value of at least 9 was required to produce stable and homogeneous composite hydrogels. The deprotonated form of the PEI (with more primary and secondary unshared electron pairs of nitrogen available) was more reactive with the amide groups of the polymer.

Therefore, when designing a conformance-improvement treatment, it is important to take into account factors that can lead to changes in the pH value of the crosslinker or the gelant (e.g., gelant mixing with reservoir fluids, reaction/adsorption of the gelant components with the formation, and presence of sour gases such as CO_2_ and H_2_S).

#### 2.3.5. Clay Concentration

Gelants prepared with a higher clay concentration presented longer gelation times and slightly greater storage modulus, and thus complex modulus (final gel strength). In the absence of clay, the polymer chains were randomly crosslinked by PEI, forming conventional hydrogel structures. However, when clay was added to the gelant formulation, the nonionic functional groups of the polymer chains (acrylamide and NVP) adsorbed onto the bentonite particles through strong hydrogen bonds between the amide protons of the acrylamide and the carbonyl oxygen of the NVP and the silanol (–Si–OH) and aluminol (–Al–OH) groups of the clay platelets, reducing the number of crosslinking sites effectively available to react with PEI, thereby delaying the gelation reactions [39].

Moreover, the adsorption of the polymer chains onto the clay particles reinforced the hydrogel network, mainly through the formation of clay networks and the diffusion of polymer chains through clay tactoids (restricting their mobility). Within the composite hydrogel structure, the clay particles acted as active fillers, contributing to slightly increase the elastic modulus of the gel [23,51,52,53].

With formulation F4 as reference, when the clay concentration increased twofold to 1.6 wt % (F5), the viscosity of the gelant increased to 26 mPa·s, the gelation time was delayed to 15 h, and the final gel strength slightly increased to G*_e_ ~10 Pa (Sydansk code F). On the other hand, when no clay was added to the formulation (F3), the viscosity of the gelant decreased to 19 mPa·s, the gelation time decreased to 11 h, and the final gel strength decreased to G*_e_ ~6.5 Pa (Sydansk code F).

Furthermore, the final strength (complex modulus) of the composite hydrogels remained unchanged during the thermal aging process for a longer period than the conventional hydrogels (with no clay), indicating a greater thermal stability of these hybrid clay/polymer structures (~6 months).

In the presence of high salinity/hardness brines (e.g., formation water), the composite hydrogels exhibited less syneresis than the conventional hydrogels. The higher resistance to volume shrinkage was attributed to the interaction of the clay tactoids with polymer segments throughout the gel network.

Although the addition of clay to the gelant formulation increased the gelation time, the thermal stability and the syneresis resistance of the composite hydrogel, when added above a certain concentration (1.6 wt %) the proper dispersion of the clay particles between polymer chains did not take place [23,54].

#### 2.3.6. Clay Type

**Figure 5:**
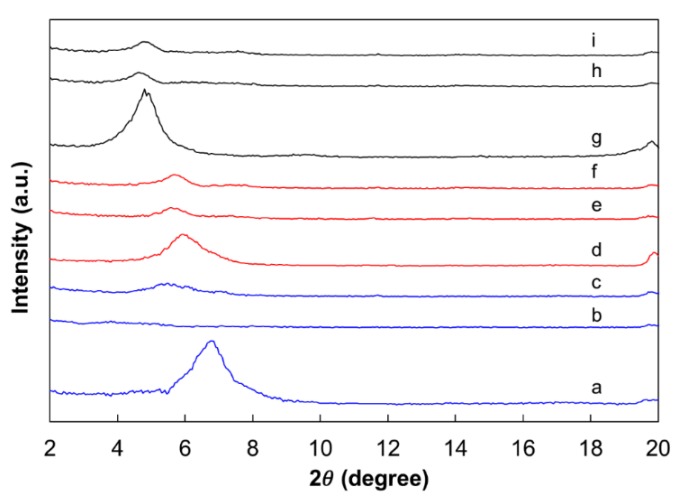
X-ray diffraction spectra of natural clays and hydrogel samples prepared in distilled or field water at 85 °C, with 0.6 wt % polymer, 0.5 wt % PEI, and 0.8 wt % clay (F4). **a**, sodium bentonite *in natura*; **b**, sodium bentonite nanocomposite (distilled water); **c**, sodium bentonite composite (brine); **d**, polycationic bentonite *in natura*; **e**, polycationic bentonite composite (distilled water); **f**, polycationic bentonite composite (brine); **g**, organically modified bentonite *in natura*; **h**, organically modified bentonite composite (distilled water); and **i**, organically modified bentonite composite (brine).

**Figure 6:**
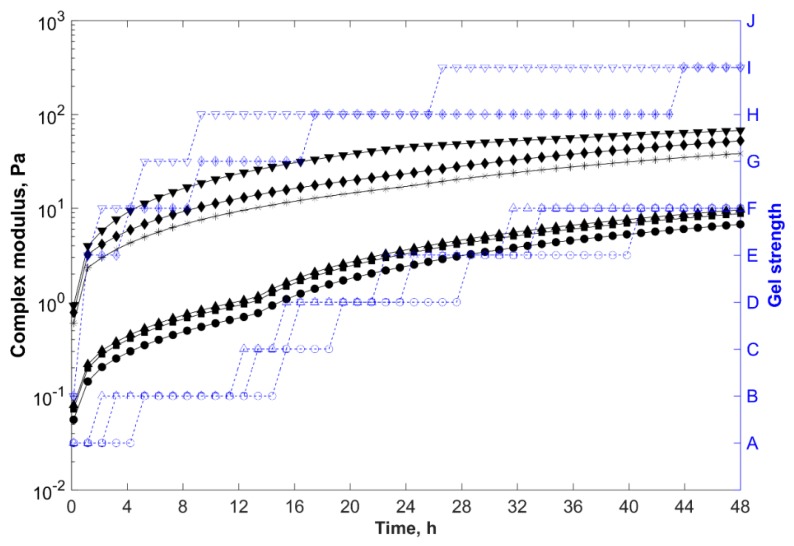
Complex modulus (─) and gel strength (- -) versus time for samples prepared in distilled or field water at 85 °C, with 0.6 wt % polymer, 0.5 wt % PEI, and 0.8 wt % clay of different types (F4). 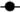

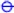
, organically modified bentonite composite (brine); 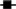

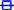
, polycationic bentonite composite (brine); 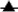

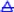
, sodium bentonite composite (brine); 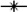

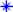
, organically modified bentonite composite (distilled water); 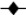

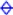
, polycationic bentonite composite (distilled water); and 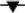

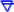
, sodium bentonite nanocomposite (distilled water).

In the X-ray diffraction spectra (Figure 5), the sodium bentonite, polycationic bentonite, and organically modified bentonite had strong peaks at 2θ of around 6.8°, 5.9°, and 4.8°, respectively, which corresponded to basal spacing of approximately 13, 15, and 18 Å. According to the results shown in Figure 5, it is possible to suggest the morphology of the formed hydrogels. The sodium bentonite formed nanocomposite hydrogels with exfoliated structure in distilled water, with bentonite platelets dispersed in the polymer matrix at nanoscale (Figure 6). This resulted in stronger interactions between the polymer chains and clay layers, resulting in greater final gel strength. However, it reduced the gelation time, probably because the individual clay platelets acted as additional crosslinking sites for the polymer chains (Figure 6).

On the other hand, the sodium bentonite formed partially intercalated structures in field brine (with divalent ions)—in which the polymer chains were inserted into the interlayer space of the clay particles, and were weakly bound to the hydrated interlayer cations and to the silicate layers by van der Waals forces and hydrogen bonds, respectively (Figure 5)—shifting the 2θ peak to lower angles (5.45°), corresponding to basal spacing of approximately 16 Å.

The polycationic bentonite and organically modified bentonite probably formed microcomposite structures both in distilled water and field water. The XRD spectra of these composite hydrogels looked essentially the same as those obtained for the clay powders, which indicated that the polymer chains did not penetrate between the clay layers (there was no shifting of the X-ray d-spacing), interacting only with the external surfaces of the tactoids or aggregates of tactoids (Figure 5) [52,55,56,57,58,59,60,61,62,63,64].

These morphologies reduced the interactions between polymer chains and clay particles when compared to fully exfoliated nanostructures. As a result, these composite hydrogels presented longer gelation times and weaker gel strengths when compared to the sodium bentonite nanocomposite hydrogel prepared in distilled water.

The (nano)composite hydrogel prepared in distilled water with sodium bentonite, polycationic bentonite, and organically modified bentonite presented viscosities around 420, 310, and 205 mPa·s; gelation times <1 h; and final gel strengths (G*_e_) around 67, 52, and 38 Pa (with Sydansk code = H), respectively. Nevertheless, in field water with high salinity/hardness, the superior performance of the sodium bentonite over the other two clays was not observed. The gelling systems prepared with pure commercial clays Cloisite Na^+^ and Cloisite 30B presented rheological behavior, gelation time, and final gel strength similar to the one formulated with low-cost natural polycationic bentonite (viscosity ~ 24 mPa·s, gelation time ~ 13 h, G*_e_ ~ 9 Pa, and Sydansk code = F).

#### 2.3.7. Temperature

During conformance treatment, gelling systems were exposed to several temperature gradients. First, during preparation in the topside facilities, the gelants were exposed to variations in the ambient temperature. Soon after being injected into the reservoir, the gelants cooled down the formation surrounding the wellbore, leading to temperatures much lower than the reservoir temperature. In the absence of convective flow, the temperature to which the gelant was exposed rose slowly due to the low thermal conductivities of the reservoir rocks and fluids. After an appropriate reservoir shut-in time, the gelants finally reached the reservoir temperature.

**Figure 7:**
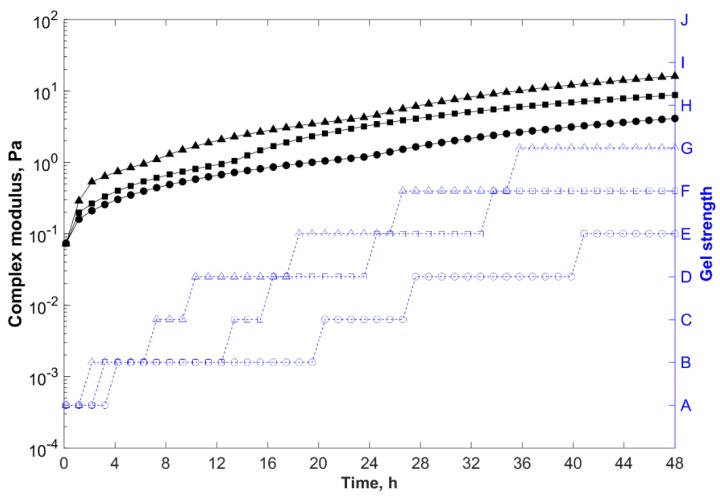
Complex modulus (─) and gel strength (- -) versus time for samples prepared in field water with 0.6 wt % polymer, 0.5 wt % PEI, and 0.8 wt % clay (F4), at different temperatures. 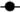

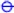
, 65 °C; 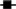

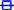
, 85 °C; and 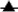

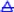
, 105 °C.

Figure 7 shows the gelation kinetics of gelling systems at different temperatures. Gelants prepared at higher temperatures presented shorter gelation times and greater storage modulus, and thus complex modulus (final gel strength).

At room temperature (25 °C), all studied gelant systems prepared in desulfated seawater did not gel for six days. This behavior was highly desirable for formulations applied in conformance treatments because if these systems were mixed in the topside facilities, and could not be promptly injected into the formation due to any operational problem, the gelants would not gel in the mixing tank, and would still be available to be pumped into the target zone for approximately one week.

For reservoir temperatures above 65 °C, since the gelation reactions were endothermic, the greater the temperature was, the faster was the gelation kinetics (crosslinking reaction between the polymer and the crosslinker) and the stronger was the final gel strength.

Higher temperatures led to an increase in the molecular mobility of polymer chains, so more crosslinks could be formed. Furthermore, higher temperatures can cause thermal hydrolysis of acrylamide groups (increasing the acrylate moieties in the polymer backbone), accelerating the crosslinking reactions due to the greater accessibility of the acrylamide groups by PEI resulting from the electrostatic repulsions between the newly charged carboxylate groups and the sulfonate groups [42,43,65,66].

With formulation F4 crosslinked at 85 °C as reference, when the temperature increased to 105 °C, the gelation time decreased to 7 h, but a strong gel was formed after 48 h (with Sydansk code = G and G*_e_ of 16 Pa).

Therefore, to slow down the gelation kinetics of these gelling systems for in-depth conformance treatment of hot reservoirs, it is necessary to use gelants with lower crosslinker and greater clay concentrations, add chemical retardants to the formulation, or pre-flush the treated zone to cool it before the gelling system is injected [15].

#### 2.3.8. Salinity (Total Dissolved Solids—TDS)

During conformance treatment, the gelling systems were exposed to several salinity gradients. First, in the topside facilities gelants were prepared by mixing the polymer and crosslinker with injection water. In offshore applications, desulfated seawater was generally used for this purpose. Fresh water or any other production brine available may also be used in gelant formulations, especially in onshore applications. After being injected into the reservoir, the gelling systems came progressively into contact with reservoir fluids, and their original water could be partially or totally replaced by the formation water.

For conformance treatments within the well or in the formation near the wellbore (at distances <15 m), the gel setting may occur with the injection water salinity. For in-depth conformance treatments, the gel setting within the reservoir generally occurs in a brine with intermediate salinity between that of the injection water and the connate water, represented in this study by the field water composition. Gel setting may also occur under high connate water salinity if the gelant reaches an aquifer during the treatment.

The gelation time and the final gel strength were strongly dependent on the total salt content (ionic strength) of the water in which the gelants were prepared (e.g., injection water) and the hydrogels were formed (e.g., field water) [16,19,52].

**Figure 8:**
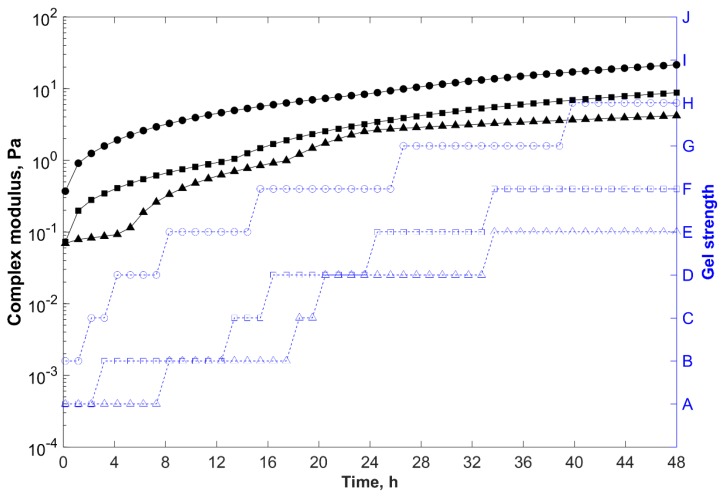
Complex modulus (─) and gel strength (- -) versus time for samples prepared at 85 °C, with 0.6 wt % polymer, 0.5 wt % PEI, and 0.8 wt % clay (F4), in water with different total dissolved solids (TDS). 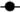

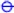
, 33,489 mg/L (injection water); 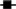

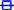
, 56,012 mg/L (field water); and 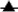

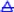
, 123,582 mg/L (reservoir water).

Figure 8 shows the gelation kinetics of gelling systems with different salinities. Gelants prepared with less saline brines presented shorter gelation times and greater storage modulus, and thus complex modulus (final gel strength).

The salts interacted directly with the clay particles and the macromolecules binding to the hydrophilic charged groups of the filler and polymer (e.g., sulfonate and formed carboxylate groups). As a result, these negatively charged groups were shielded by the metal ions, especially by divalent cations. This screening effect caused the polymer chains to shrink in a random coil configuration (smaller hydrodynamic volume), and the clay tactoids to aggregate. Thereby, the clay/polymer interactions were weakened, and potential crosslinking sites of the polymer chain were not as accessible to react with PEI, resulting in longer gelation time and weaker composite hydrogels [16,42].

With formulation F4 crosslinked with field water (56,012 mg/L TDS) as reference, when the salinity increased to 123,582 mg/L (connate water), the gelation time increased to 18 h and the gel strength after 48 h declined to G*_e_ ~ 4 Pa (with Sydansk code = E).

Therefore, for the permeability-reducing treatment of conformance problems in which high salinity/hardness connate water is present (such as when 2D water is coning up from an aquifer underlying the oil reservoir through a vertical fracture or other reservoir irregularity of a vertical wellbore), higher polymer loadings and/or pre-flushing the treated zone with fresh water might be necessary. Furthermore, in cases in which wide fractures or large vugs are present, the mechanical strength and plugging characteristics of the gel can be increased by adding particulate matter (e.g., sand, fibers, nutshells, clay, etc.) [1].

#### 2.3.9. Salt Type

**Figure 9:**
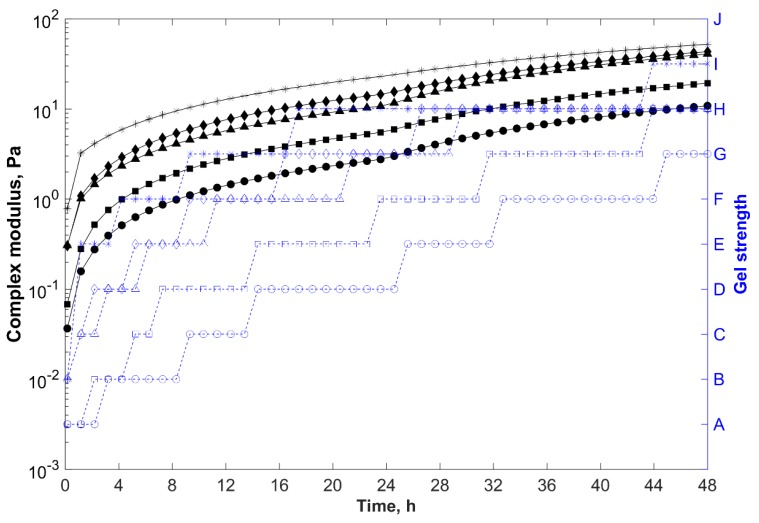
Complex modulus (─) and gel strength (- -) versus time for samples with 0.6 wt % polymer, 0.5 wt % PEI, and 0.8 wt % clay (F4), prepared at 85 °C in distilled water and 1.0 wt % saline solution of different salts. 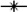

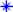
, distilled water; 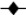

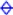
, KCl; 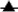

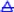
, NaCl; 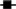

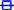
, MgCl_2_; and 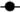

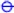
, CaCl_2_.

In order to better understand the influence of the charge and the size (radius) of the cations on the gelation kinetics of the acrylamide-based systems, four cations of chloride salts (with the same anion) were evaluated—sodium (Na^+^), potassium (K^+^), calcium (Ca^2+^), and magnesium (Mg^2+^), as shown in Figure 9.

As previously discussed, for the negatively charged sites of the polymer (formed carboxylate and sulfonate groups) and clay particles, the mono- and divalent cations had an electrostatic shielding effect, which reduced the electrostatic repulsion between the anionic groups of the polymer chain and clay tactoids.

Furthermore, the mono- and divalent cations interacted with the uncharged groups (acrylamide and NVP) of the polymer chain, and with the deprotonated PEI due to attractive forces between cations and polymer dipoles. The most likely binding sites were the amide carbonyl oxygen of the polymer chain and the amine nitrogen of PEI, which carried a partially negative charge because of the resonant states of dipoles [67,68].

The samples prepared in KCl, NaCl, MgCl_2_, and CaCl_2_ brines showed, respectively, viscosities of 45, 42, 38, and 31 Pa; gelation times around 0.5, 1, 5, and 10 h; as well as final gel strengths of approximately 43, 41, 19, and 11 Pa.

The valence, hydration shell strength and size of the cations affected the interaction with hydrophilic sites of the polymer, PEI, and clay particles, as well as with surrounding water molecules, which influenced the viscosity, gelation time, final gel strength, and clay dispersion in the acrylamide-based systems studied. These results were in accordance with other findings reported in the literature [43,68,69].

Cations with greater charge densities (ionic charge/ion size)—higher valences and smaller radii —delayed the gelation and decreased the viscosity, the clay dispersion, and the final gel strength of acrylamide-based systems more intensely. Furthermore, cations with stronger hydration shells and same valence exerted a weaker influence on the viscosity, clay coagulation, and gelation kinetics of the gelling systems.

The cation valence (ion charge) was the main parameter that influenced the viscosity, gelation kinetics, and clay dispersion within the hydrogel. Divalent cations (Ca^2+^ and Mg^2+^), with higher ionic charges (higher valences), had a more pronounced effect on the gelation delay, as well as on the reduction of the viscosity, clay dispersion, and final gel strength, than monovalent cations (Na^+^ and K^+^).

Moreover, for cations with the same valence and weaker hydration shells, the ion size appeared to be the parameter responsible for the variation in viscosity and gelation kinetics of the gelling systems. For instance, in aqueous solutions, monovalent ions (Na^+^ and K^+^), with loose hydration layers constantly breaking–reforming, bound directly to hydrophilic sites of the polymer, PEI, and clay particles, with minor interference of the hydration shell. As a result, the dehydrated sodium cations (Na^+^), with smaller radius (ionic radius of 0.95 Å), and thus with greater charge densities, delayed gelation and reduced the viscosity and the final gel strength more significantly than the dehydrated potassium cations (K^+^) (ionic radius of 1.33 Å) [43,70,71,72].

**Figure 10:**
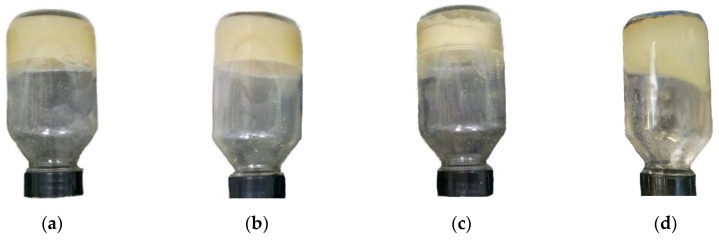
Acrylamide-based polymers, PEI, and bentonite hydrogels prepared at 85 °C in 1.0 wt % brine of (**a**) NaCl, (**b**) KCl, (**c**) MgCl_2_, and (**d**) CaCl_2_.

Also, the monovalent cations of the solution (Na^+^ and K^+^) partially exchanged with the interlamellar ions of the polycationic bentonite, improving the particle dispersion within the hydrogel (Figure 10a,b) due to partial exfoliation of the clay platelets, contributing to reduce the gelation time, and also increased the viscosity of the gelant and the final strength of the composite hydrogels.

In contrast, with divalent cations, more strongly solvated by water molecules than the monovalent cations, the viscosity, gelation kinetics, and clay dispersion within the hydrogel were influenced by the hydration shell strength of the ions.

Calcium ions (Ca^2+^), which have relatively loose hydration layers that can be easily dehydrated by the electrostatic attraction between cation and negative groups of polymer chains and/or clay particles, were weakly influenced by the hydration shell during cation–polymer–clay interactions. On the other hand, magnesium ions (Mg^2+^), which have stable (difficult to dehydrate) hydration shells, interacted more weakly and indirectly with the polymer chains and clay particles than Ca^2+^ ions because the dense and strongly adhered hydration layer prevented the Mg^2+^ cations from approaching the polymer chains and clay tactoids. As a result, the hydrated calcium cations (Ca^2+^), with smaller radius (hydrated radius of 4.1 Å), and thus with greater charge densities, delayed gelation and reduced the viscosity and the final gel strength more significantly than the hydrated magnesium cations (Mg^2+^) (hydrated radius of 4.3 Å) [43,70,71,73].

Furthermore, the Ca^2+^ ions acted as coagulants, bonding to the negatively charged clay particles, increasing their settling behavior, and thus the heterogeneity of the formed composite hydrogel (Figure 10d). In contrast, the Mg^2+^ ions, due to their greater hydration shells, did not bind to the clay particles, acting instead as ionic crosslinkers in the medium, complexing with sulfonate (–SO_3_^−^) and carboxylate (–COO^−^) groups of the polymer chains [74], and increasing the viscosity, strength, and instability (syneresis) of the hydrogel (Figure 10c).

Besides varying with the charge density and the hydration shell strength of the cations, the magnitude of the salt-type influence on the increase of the gelation time, as well as on the reduction of the viscosity, final gel strength, and clay dispersion within the acrylamide-based hydrogels followed the Hofmeister series—Ca^2+^ > Mg^2+^ > Na^+^ > K^+^.

### 2.4. Overview of the Influence of the Different Parameters Studied on the Gelation Time and Gel Strength

Table 3 presents an overview of the qualitative influence of the different parameters studied on the gelation time and final gel strength of AMPS–NVP–acrylamide–PEI–clay based systems.

## 3. Conclusions

The injectivity, propagation, gelation time, and final gel strength of AMPS–NVP acrylamide terpolymer/PEI/bentonite were strongly influenced by the gelant formulation and field conditions (temperature and salinity/hardness). The greater the polymer, the crosslinker concentration, the PEI molecular weight or the PEI initial pH value in the gelant, or the reservoir temperature, the shorter was the gelation time and the greater was the storage modulus, and thus the complex modulus (final gel strength) of the system. On the other hand, the greater the salinity and/or hardness of the medium, the longer was the gelation time and the weaker was the final gel strength.

The polymer concentration, salinity/hardness, and temperature were the parameters that most strongly affected the gelation time, and hence how deep the gelant can penetrate into the formation, as well as how long the reservoir shut-in time should be during conformance treatment. Moreover, the final gel strength was mainly controlled by the polymer concentration, divalent ion concentration, and crosslinker concentration and molecular weight. Nevertheless, the higher the crosslinker concentration or molecular weight, the higher was the composite hydrogel syneresis.

The addition of clay to the gelant formulation increased the gelation time, thermal stability, and syneresis resistance of the composite hydrogel, and slightly increased the final gel strength.

Of the samples evaluated, formulation F4 prepared with polymer and PEI concentrations below 1 wt %, low-cost polycationic natural bentonite, and PEI with $\bar{M_{w}}$ of 70,000 kg/kmol and initial pH value around 11: (i) presented suitable rheological behavior to ensure good injectivity and propagation in porous media (moderate pseudoplastic fluid and viscosity ~24 mPa·s at 7.3 s^−1^); (ii) showed sufficient gelation time for the treatment of targeted zones located far from the wellbore (e.g., ~7 h); and (iii) formed strong composite hydrogels under subsurface conditions (G*_e_ ~ 16 Pa and Sydansk scale G, after 48 h at 105 °C), presenting good blocking ability characteristics, low syneresis, and good long-term stability (~3 to 6 months).

Therefore, the use of high-molecular-weight base polymers (>1,000,000 kg/kmol) and the addition of inexpensive natural bentonites as active fillers in the formulations of OCP systems are promising for in-depth conformance treatments under harsh conditions (high temperature, high salinity, and high hardness).

## 4. Materials and Methods

### 4.1. Materials

The gelling systems were prepared using a commercial sample of acrylamide, 2-acrylamido-2-methylpropane sulfonic acid, and *N*-vinyl-2-pyrrolidone terpolymer (PAM–AMPS–NVP), supplied by SNF Inc. (Riceboro, GA, USA). Three samples of bentonites were tested as fillers—a Brazilian natural polycationic bentonite (with calcium and magnesium exchangeable cations), supplied by Bentonit União do Nordeste Ltda (São Paulo, Brazil); a sodium bentonite (Cloisite Na^+^); and an organically modified bentonite (Closite 30B), both supplied by Southern Clay Products (Gonzales, TX, USA). Three samples of branched polyethylenimine (PEI) with pH value around 11, and weight average molecular weights ($\bar{M_{w}}$) of 10,000, 70,000, and 750,000 kg/kmol, supplied by Polysciences Inc. (Warrington, PA, USA), were used as crosslinkers. The pH value of PEI was adjusted to 2, 7, or 9 by adding a few drops of 1.0 M HCl solution when necessary. Oilfield brines with representative composition of the salinity/hardness found in the gelant during preparation (injection water), gel setting within the reservoir (field water), or contact with an aquifer (connate water), as shown in Table 4, were prepared with NaCl, KCl, CaCl_2_, and MgCl_2_·6H_2_O, supplied by Vetec Química Fina Ltda (Duque de Caxias, Brazil).

### 4.2. Characterization of the Samples

The chemical composition of the polymer sample was determined by ^13^C-NMR spectroscopy with a Varian Mercury VX 300 MHz spectrometer (International Equipment Trading Ltd., Mundelein, IL, USA), at a frequency of 75.4 MHz, acquiring about 48,000 transients at 40 °C. The spectrum was calibrated using sodium 3-(trimethylsilyl)-2,2′,3,3′-tetradeuteropropionate (TSP) methyl as an internal reference (zero ppm). The polymer solution was prepared in heavy water (D_2_O).

The weight average molecular weight ($\bar{M_{w}}$) and the polydispersion (PD) of the acrylamide-based sample were assessed by gel permeation chromatography (GPC, Malvern Panalytical, Malvern, UK) with a Max VE 2001, GPC Solvent/Sample Module, Viscotek chromatograph equipped with differential refractometer, viscometer, ultraviolet, and light scattering detectors, and three columns in series with an exclusion limit of 2 × 10^7^.

The microstructure of the clays and the acrylamide-based composite hydrogels prepared in distilled water and field brine were analyzed by X-ray diffraction with a Rigaku Ultima IV diffractometer (Rigaku, Tokyo, Japan), with Cu-filtered Kα radiation, at 40 kV and 20 mA. The samples were scanned in 2θ ranges from 1° to 20° with a step size of 0.05°.

### 4.3. Gelling System Preparation

Polymer solutions and clay dispersions were prepared by slowly mixing the polymer and clay in distilled water or oilfield brines (Table 4) under moderate magnetic stirring (330 rpm) for 72 h at room temperature (25 °C). Then the clay dispersions were added to the polymer solutions, and the mixtures were stirred further for 1 h. Finally, the crosslinking agent (PEI) was added, and the systems were stirred for another 15 min before conducting bottle tests and rheological tests.

Initially, all gelling systems (Table 5) were prepared using Brazilian natural polycationic bentonite as filler and PEI with $\bar{M_{w}}$ of 70,000 kg/kmol and pH value around 11 as crosslinker in order to evaluate individually the influence of the polymer concentration, clay concentration, and PEI concentration on the viscosity of the systems prepared with injection water at 25 °C, and on the gelation kinetics and final gel strength of the systems prepared in field water (56,012 mg/L TDS) at 85 °C.

Then, additional gelling systems were prepared based on the standard composition F4 (Table 5), varying exclusively (i) the clay type (sodium bentonite or organically modified bentonite); (ii) the molecular weight of the crosslinker (10,000 kg/kmol or 750,000 kg/kmol); (iii) the pH value of the crosslinker (2, 7, or 9); (iv) the temperature during the gelation process (65 °C or 105 °C); (v) the salinity during the gelation process (0 mg/L, 33,489 mg/L, or 123,582 mg/L TDS); and (vi) the salt type of the brine during the gelation process (1 wt % NaCl, 1 wt % KCl, 1 wt % CaCl_2_, or 1 wt % MgCl_2_), while keeping the other parameters unaltered.

These additional formulations were evaluated to better understand the impact of the clay type, PEI molecular weight, and pH value on the viscosity, gelation kinetics, and final gel strength, and to assess the impact of the reservoir conditions (temperature, salinity, and salt type) on the gelation process of the systems. Only the most important results of these additional formulations are presented in Section 3, while the individual parameter altered in relation to the standard F4 composition is mentioned in the text and/or in the caption of the figures.

### 4.4. Evaluation of the Injectivity, Propagation, Gelation Kinetics, and Gel Strength

Four important parameters for conformance-improvement treatments of oil reservoirs were assessed by bottle tests and rheological tests—the injectivity and propagation of the gelling systems and the gelation time and final strength (equilibrium consistency) of the hydrogels. The measured quantities in this study were reproducible with an error of less than 10%. All measurements were repeated three times and the average values of the measured quantities were reported.

#### 4.4.1. Bottle Test

Bottle tests were performed by adding 20 mL of gelant in glass bottles with cap (60 mL). Then, the bottles were sealed and placed in an air circulation chamber with controlled temperature set to 65, 85, or 105 °C. For up to 48 h, at varying time intervals, the bottles containing the gelling formulations were vertically inverted and gel strength codes were assigned to the samples according to the behavior (flowability) observed.

The gel strength scale A to J proposed by Sydansk [11], visually presented by Tessarolli et al. [35], was used in this study—A for no visually detectable gel formed; B for highly flowing gel; C for flowing gel; D for moderately flowing gel; E for slightly flowing gel; F for highly deformable non-flowing gel; G for moderately deformable non-flowing gel; H for slightly deformable non-flowing gel; I for rigid gel; and J for rigid ringing gel.

The gelation point of each formulation (transition from liquid to solid) was considered to occur when the gel strength code changed from B to C [75,76,77].

#### 4.4.2. Rheological Tests

Steady and oscillatory shear tests were performed with a Haake MARS 60 rheometer using cone-plate (C60) and coaxial cylinder (CC25) geometries, respectively (Table 6).

The storage modulus (G’) represents the portion of the stress energy that is temporarily stored during the test, but can be recovered afterwards. The loss modulus (G”) represents the portion of the stress energy that is used to initiate the flow and is irreversibly transformed into shear heat. The complex modulus (G*) is a rheological parameter that quantifies the total resistance (consistency) of a gelling system against the maximum applied strain. The G* value is related to the storage modulus (G’) and loss modulus (G”) according to Equation (4).

(4) $$G^{*}=\sqrt{{G^{\prime }}^{2}+G^{\prime \prime }{}^{2}}$$

## Figures and Tables

**Table 1:** Assignment of the chemical shift of the carbons of the polymer sample.

Carbon Index	Chemical Shifts (ppm)	Assignment of the Peaks
C11	181 to 179	Carbonyl-carbon (C=O) of the amide group (−CONH_2_)
C9	179 to 177	Carbonyl-carbon (C=O) of the NVP ring
C10	177 to 175	Carbonyl-carbon (C=O) of the AMPS group
C8	61 to 59	Methylene-carbon (–CH_2_) of the AMPS group
C7	56 to 53	Carbon linked to CH_2_–SO_3_–Na^+^ of AMPS
C6	51 to 50	Methylene-carbons (–CH_2_) of the NVP ring located further to the carbonyl carbon
C5	45 to 41	Methine-carbons (–CH) of the polymer backbone attached to the amide (–CONH_2_), AMPS-Na or NVP groups
C4	39 to 34	Methylene-carbons (–CH_2_) of the polymer backbone attached to the amide (–CONH_2_), AMPS-Na or NVP groups
C3	34 to 33	Methylene-carbons (–CH_2_) of the NVP ring located close to the carbonyl carbon
C2	30 to 27	Methyl-carbons (–CH_3_) of the AMPS group
C1	20 to 19	Methylene-carbons (–CH_2_) of the NVP ring

**Table 2:** Chemical composition, molecular weight, and polydispersion of the polymer sample.

Sample	AM Moieties (%)	AMPS Moieties (%)	NVP Moieties (%)	$\bar{M_{w}}$ (kg/kmol)	PD
AMPS–NVP–AM	48	30	22	2 × 10^6^	1.02

**Table 3:** Overview of the influence of the different parameters on the gelation time and gel strength.

Parameters		Gelation Time	Final Gel Strength
**Gelling System Formulation**	↑ polymer concentration	↓↓	↑↑
	↑ crosslinker concentration	↓↓	↑↑
	↑ clay concentration	↑	↑
**Crosslinker Properties**	↑ crosslinker molecular weight ↑ crosslinker initial pH value (gelant pH value)	↓↓ ↓↓	↑↑ ↑↑
**Clay Properties**	↑ divalent exchangeable cations in the intralamellar space	↑	↓
**Well or Reservoir Conditions**	↑ temperature	↓↓	↑↑
	↑ salinity	↑↑	↓↓
	↑ hardness	↑↑	↓↓

↑, Slight increase; ↓, slight decrease; ↑↑, strong increase; and ↓↓, strong decrease.

**Table 4:** Composition of typical oilfield brines.

	Concentration (mg/L)		
Ions	Desulfated Seawater (Injection Water)	Field Water (Gel Setting Water Within the Reservoir)	Formation Water (Connate Water)
Sodium (Na^+^)	11,589	18,736	40,177
Potassium (K^+^)	225	943	3098
Calcium (Ca^2+^)	341	1430	4696
Magnesium (Mg^2+^)	677	669	646
Chloride (Cl^−^)	20,655	34,232	74,963
Total Dissolved Solids, TDS	33,489	56,012	123,582

**Table 5:** Gelling system formulations prepared with PAM–AMPS–NVP terpolymer, polycationic bentonite, and PEI with 70,000 kg/kmol and pH value of 11 in injection water or field water.

Formulations	Polymer (wt %)	Clay (wt %)	PEI (wt %)
F1	0.2	0.8	0.5
F2	0.6	0.8	0.0
F3	0.6	0.0	0.5
F4	0.6	0.8	0.5
F5	0.6	1.6	0.5
F6	0.6	0.8	1.0
F7	1.0	0.8	0.5

**Table 6:** Rheological tests applied to evaluate the gelling systems.

Test	Objective	Experimental Condition
Steady shear test with shear rate sweep	Evaluate the rheological behavior and the injectivity of the gelling systems based on the return curve of the samples.	Shear rate: 0.1 to 1000 s^−1^ Temperature: 25 °C
Oscillatory shear test with strain sweep	Determine the linear viscoelasticity region (LVR) of gelling systems and hydrogels. The LVR delimits the critical deformation that can be applied to the sample in order to ensure that its structure is not altered.	Strain: 0.01 to 100; Frequency: 0.1 Hz; Temperature: 65, 85, or 105 °C
Oscillatory shear test with time sweep (at constant strain and frequency)	Monitor the evolution of the storage modulus (G’), loss modulus (G”), and complex modulus (G*) as a function of time without any mechanical disturbance or destruction of the elastic structure of the hydrogel. The gelation time of each sample was determined when G* started to increase rapidly (inflection point).	Strain: 0.1 (in the LVR for all tested samples); Frequency: 0.1 Hz; Temperature: 65, 85, or 105 °C; Time: 48 h
